# Do Changes in Welfare and Health Policy Affect Life Satisfaction of Older Citizens in Europe?

**DOI:** 10.1155/2017/7574040

**Published:** 2017-09-25

**Authors:** Marinela Olaroiu, Ioana Dana Alexa, Wim J. A. van den Heuvel

**Affiliations:** ^1^Foundation Research and Advice in Care for Elderly (RACE), Heggerweg 2a, 6176 RB Spaubeek, Netherlands; ^2^Faculty of Medicine, University of Medicine and Pharmacy “Grigore T. Popa”, 16 University Street, 700115 Iasi, Romania; ^3^University Medical Centre, University of Groningen, P.O. Box 30001, 9700 RB Groningen, Netherlands

## Abstract

**Objectives:**

Ageing of societies causes serious political concerns on well-being of old citizens and care for the (frail) old. These concerns increased with the economic crisis of 2008. In European countries policy measures were taken to deal with the consequences of this crisis. This study explores the possible effects of these measures on life satisfaction of older citizens.

**Methods:**

Life satisfaction was assessed through international surveys in 2007 and 2013 and changes in societal conditions, using eight indicators on demography, welfare, and health, are assessed in 31 European countries in 2006 and in 2014. Data are standardised and based on official, national surveys and statistics.

**Results:**

The former found that U-shape relationship between age and life satisfaction disappeared after the crisis. Negative changes in social protection and care arrangements, taken after the economic crisis, are related to low life satisfaction in old citizens.

**Conclusions:**

Various societal conditions deteriorated in 2014 as compared to 2006. Policy measures, taken due to the 2008 economic crisis, have changed societal conditions and affected life satisfaction of older citizens negatively. In countries with a rudimentary structure of health and welfare provisions old citizens could not cope with the imposed policy measures.

## 1. Introduction

For some decades, ageing in Europe causes serious political concerns on well-being and social participation of old citizens as well as on care for the (frail) old and personnel to take care for them. These concerns increased due to the economic crisis of 2008 and are related: health and need for (long-term) care affect well-being and social participation and vice versa [1–4]. Therefore, in most European countries policy measures were proposed and taken to influence consequences of the economic crisis and ageing [5, 6]. Measures included new pension regulations, changing arrangements for long-term care and for access to social services, but the effects of such measures on society and life of citizens are unknown. This study explores which societal measures, related to the economic crisis and ageing, may have affected the life satisfaction of older citizens. Understanding the effect of such measures on life satisfaction of older citizens could inform policymakers about future measures to get a better life for old citizens [7].

Research on quality of life and life satisfaction focuses mainly on individual determinants such as age, income, marital status, health status, physical limitations, social contacts, and social participation of citizens, whose determinants mostly show a statistically significant relationship with life satisfaction [8–12].

Besides individual characteristics, the context in which people live should be taken into account, because life satisfaction is strongly context related and also depends on social comparison [13, 14]. Therefore, some researchers argue to use vignettes to assess life satisfaction to correct for the so-called differential item functioning, a bias in self-reports caused by differences in personal and sociocultural context [15]. Although a useful method, it does not take into account societal changes over time, including cohort effects, affecting life satisfaction [16, 17]. Societal changes may have a wide effect, that is, influencing the life of many people in various countries (e.g., World War II, mass migration, or an economic crisis), while others have an effect on people living in specific regions or countries (e.g., an earthquake or national legislation). Comparative studies between countries have shown the influence of specific factors, such as national income, age composition, life expectancy, and welfare provisions, on life satisfaction at societal, national level [18–21]. Such studies may show “how data on well-being can help policymakers identify the groups and countries that are bearing the brunt of the economic crisis, as well as those that are holding out better than expected, and provides a new layer of evidence to aid policy decisions.” [20].

Comparative research on life satisfaction worldwide shows a U-shape between life satisfaction and age, supposing a major influence of ageing itself. Young age (16–40) groups show relatively high life satisfaction, which decreases in middle age (40–65) groups but increases again in older age (65 and over) groups [12, 22, 23]. Such U-shape is found to be rather consistent [24], but it does not apply to all countries [3, 25, 26], indicating that specific events or societal changes may affect it in different ways. This may also apply to the economic crisis, starting in 2008.

Therefore, our premise is that this economic crisis has affected the U-shape relationship between age and life satisfaction in European countries. The first research question is, has this U-shape changed and, if so, in which direction and in which European countries?

The second research question is, which societal conditions, changed by measures due to the economic crisis of 2008, are related to life satisfaction of older citizens in Europe. We focus on older citizens because these citizens may be more vulnerable to such societal changes and therefore are an important “target group” for policy measures [3]. To answer the second question one has to look at differences in changes between countries [20].

Analyses on life satisfaction at national/country level use standardised national data (e.g., Gross Domestic Product (GDP), age dependency ratio, or life expectancy) and aggregate individual data (e.g., percentage of persons with long-standing illnesses or % of unmet health care needs). A comparative study of 27 European countries, describing changes in “a full range of subjective well-being” between 2007 and 2011 in all adult citizens, shows that GDP and percentage of people with disabilities are related to well-being. However, subjective well-being over time increased marginally and did not apply to all countries, indicating that national policy or culture may make a difference [20]. Although it was expected that the economic crisis of 2008 would show some effect on life satisfaction, one may argue that the time frame was too short to see such effects. A recently published study analyses changes in life satisfaction in 24 European countries between 2002 and 2012, also taking into account recent, mainly income related societal changes, including the economic crisis of 2008 [21]. Income related indicators do affect life satisfaction mostly, but their effects are not uniform over European countries. However, it was stated that “economic crises tend to be followed by crises in happiness.” Our study focuses on more detailed changes, like change in life expectancy, pension, health status, and quality of care, that is, especially on changes important for older people.

## 2. Methods

To “see” effects of policy measures, related to the 2008 crisis, on “life satisfaction” one has to wait till such measures are implemented in practice and experienced by citizens. Therefore, we use the time frame between 2006 and 2014 and assessed indicators two years before the crisis was started and two years after the measures taken were fully implemented. Based on former mentioned studies, we selected societal indicators on demography, on welfare, and on health.

The research design combines changes in societal indicators over time (2006–2014) with a comparison between countries to analyse which changes in national indicators affect life satisfaction of old citizens (65 years and over) in 2013. The data are based on representative samples in each country like the data on life satisfaction or subjective health or on official, national statistics collected by Eurostat or OECD.

Life satisfaction is assessed as “the degree to which an individual judges the overall quality of his life,” to be scored on a 10-point scale between “not satisfied at all” and “fully satisfied,” using data of the EU-SILC AHM 2013 study [12]. To answer the first research question, life satisfaction of different age groups in 31 European countries is compared.

To answer the second research question we selected the following indicators to measure societal conditions, as independent variables:

  Demographic indicators: old dependency ratio (65+ to population 15–64 years) [27] and* life expectancy at birth* [28].
  Welfare indicators: % of GDP for social protection [29], % of GDP for long-term care [30], and aggregate replacement ratio, an indication for gross pension for 65–74 years old as compared to gross earnings of 50–59 years old [31].
  Health indicators: people with very good subjective health (citizens 16 years or older) [32], % of long-standing illnesses [33], and % of self-reported unmet needs in health care [34].
  SPSS 23 is used for data storage and analysis.

### 2.1. Analysis

First, we present the average life satisfaction of adult citizens in 31 European countries in 2007 and 2013.

Next, the relationship between age and life satisfaction in 31 European countries in 2013 is described; that is, is a U-shape present? The relationship between mean life satisfaction and age in 2014 is described for the following age categories: 16–24, 25–34, 35–49, 50–64, 65–74, and 75 and over. We ranged countries in four figures.

Next, the existence of statistically significant differences in mean life satisfaction of older citizens as compared to adult citizens will be tested for 2007 and 2013. Mean paired sample test for analysis of variance is used to test the difference in life satisfaction between adult citizens till 65 years and citizens 65 years and over.

Before answering the second question “do changes in demographic, welfare, and health indicators between 2006 and 2014 affect life satisfaction in older citizens in 2013?” we present the data of the eight independent indicators for each European country in 2006 and 2014 (Tables 2–4). Next bivariate Pearson correlations between differences in mean life satisfaction of citizens between 16 and 65 years versus 65 years and older and the independent indicators in 2014 are described. The mean differences of the eight indicators for 2006 and 2014 are calculated per country and tested on statistical significance. Significant differences are described. In the last analysis step, the influence of these mean differences (2006–2014) in indicators on life satisfaction of older citizens in 2013 is assessed, using linear regression analysis and enter method with collinearity (VIF) tested.

## 3. Results

The mean life satisfaction scores for all citizens (over 16–18 years) in 2007 and 2013 in the 31 countries are about the same 7,0 and 7,1, respectively (see Table 1). Overall, the tendency is that life satisfaction decreased in western European countries and increased in central-eastern European countries. However, some considerable differences exist between countries. Mean life satisfaction decreased with at least 0,5 points in Cyprus, Denmark, Estonia, and Malta but increased with at least 0,5 points in Austria, Hungary, Latvia, and Romania.

**Figure 1:**
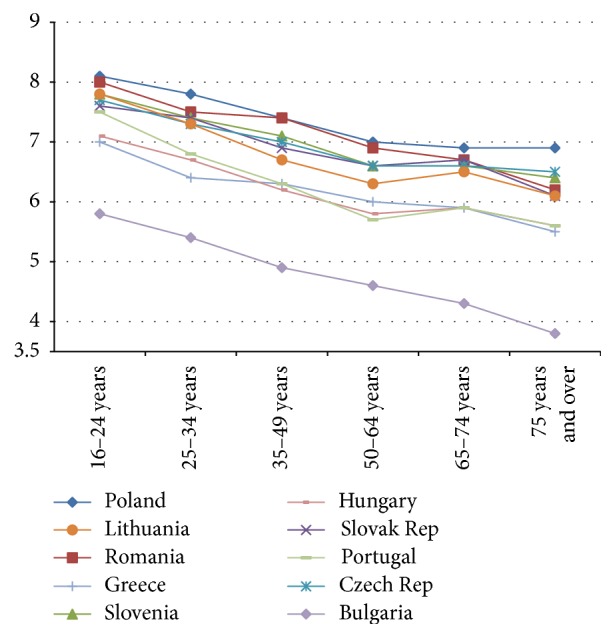
Relatively large decline in life satisfaction by age 2013.

**Figure 2:**
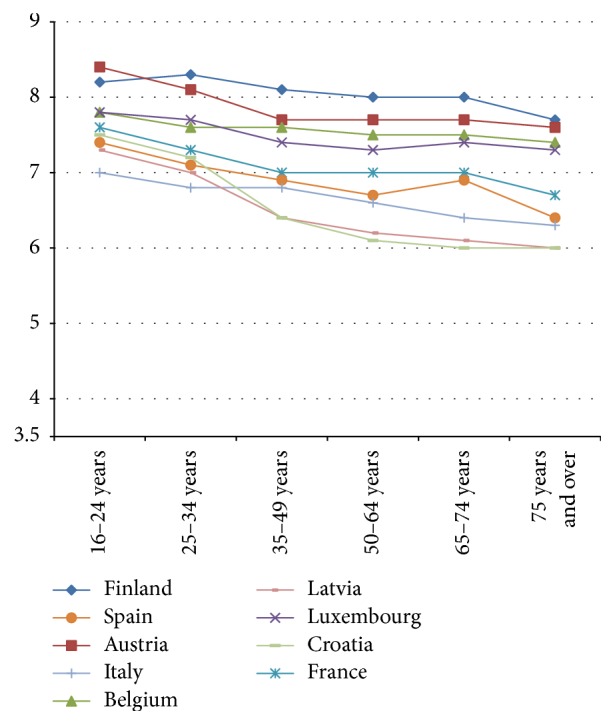
Relatively small decline in life satisfaction by age 2013.

**Figure 3:**
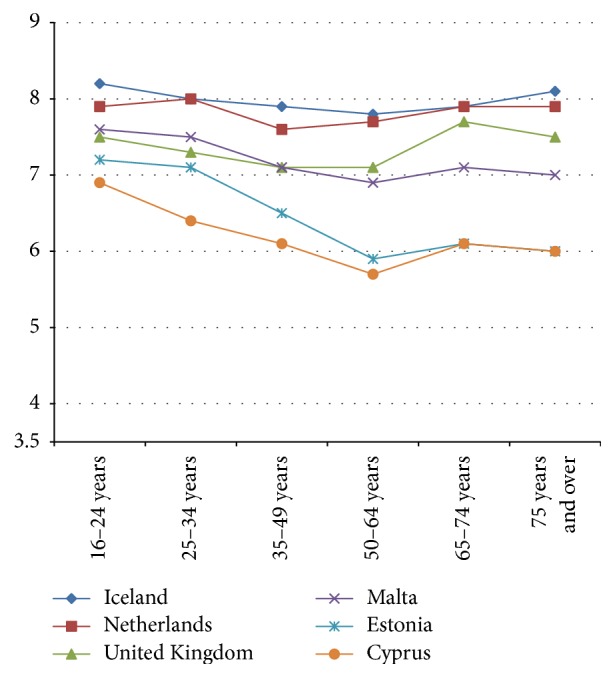
Relatively small U-shape in life satisfaction by age 2013.

**Figure 4:**
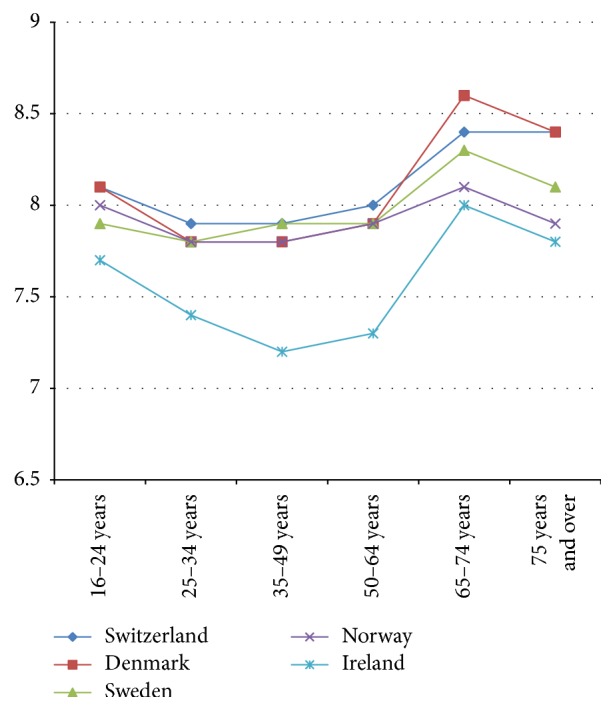
Relatively large U-shape in life satisfaction by age 2013.

The relationship between age categories and mean life satisfaction per country shows that a “U-shape” does not dominate in 2013. The majority of countries (19 out of 31) show a declining line in life satisfaction from young citizens (16–24 years) to old citizens (75 years and over) (see Figures 1 and 2). Twelve countries show more or less a U-shape (see Figures 3 and 4).

The declining gradient between age and life satisfaction is most notable in Bulgaria, Croatia, Greece, Latvia, Portugal, and Romania. In Denmark, Switzerland, Sweden, Norway, and Ireland citizens in the age group 65–74 score highest in life satisfaction, where score declines in the 75 years and over age group, with the exception of Switzerland (see Figure 4). An increase in life satisfaction at 75 years and over is only found in Iceland (see Figure 3).

No statistically significant difference in mean life satisfaction is found between the two age groups (18–64 years versus 65 years and over) in 2007. The mean score on life satisfaction in 2007 applies for both age groups, that is, 7,0.

The mean life satisfaction for citizens till 65 years is 7,2 in 2013 and for citizens of 65 years and over 6,9. Analysis of variance of mean life satisfaction scores between adult citizens of 16 to 65 years and those 65 years and over in 2013 shows a statistically significant difference (*p* = .003); that is, for old citizens the mean life satisfaction is significantly lower in 2013.

In 2013 mean differences in life satisfaction are strongly decreased in older citizens in Romania, Bulgaria, Croatia, Greece, Lithuania, Portugal, Slovenia, and Slovakia as compared to not old citizens. Increase of mean life satisfaction in older citizens as compared to young ones is rare but occurs in Denmark and Ireland.

The difference in mean life satisfaction between 2007 and 2013 is due to lower life satisfaction in older citizens in 2013.

Before answering the second research question we present the mean or proportional scores for each indicator of societal change (demographic, welfare, and health) per country (see Tables 2–4).

The demographic indicators show an increase in the old age dependency ratio in all countries (except Luxembourg) as well as in life expectancy at birth between 2006 and 2014. The welfare indicator “% of GDP on social protection” increased in all but two (i.e., Hungary and Poland) countries. This increase was relatively strong in Cyprus, Denmark, Finland, Greece, Ireland, Netherlands, and Spain. The welfare indicator “% GDP on long-term care expenditure” stayed on average the same in 2006 compared to 2014. The only decrease was in Romania; a strong increase occurred in Finland and Norway. The “aggregate replacement ratio” increased slightly between 2006 and 2014 in most European countries, but not in Austria, Estonia, Germany, Italy, and Sweden.

A quarter of citizens in the 31 European countries reported very good health in 2006 and in 2014. On average there is a slight decrease between 2006 and 2014. The score of this health indicator varies strongly between countries with low scores (<10%) in Estonia, Latvia, Lithuania, and Portugal and high scores (>40%) in Cyprus, Greece, Iceland, and Ireland. A strong decrease in subjective health is reported in Denmark and Finland. The average proportion of long-standing illness/health problems stayed about the same between 2006 and 2014 in the 31 countries as did the proportion of self-reported unmet needs for they were too expensive. Long-standing illness/health problems were more frequently reported between 2006 and 2014 in Austria, Cyprus, Greece, Malta, and Portugal and less reported in Bulgaria and Luxembourg. Self-reported unmet needs in 2006–2014 increased strongly in Greece, Ireland, Iceland, and Italy and decreased strongly in Bulgaria, Germany, Lithuania, Poland, and Romania.

The relationship between the mean scores of the eight societal indicators in 2014 and difference in life satisfaction between both age groups (16–64 versus 65 and over) in 2013 is explored by Pearson's correlations (see Table 5).

Older citizens (65+) with higher life satisfaction as compared to younger ones (16–64) live in countries, which have a high life expectancy at birth, which spend a high percentage of their GDP on social protection and long-term care in 2014, and have a high percentage of citizens in very good health.

Next, it is analysed which societal indicators changed significantly between 2006 and 2014. The following indicators show significant mean changes between 2006 and 2013: old dependency ratio (mean 3,26; *p* = .00), life expectancy at birth (mean 2,23; *p* = .00), % GDP social protection (mean 3,03; *p* = .00), and % long-standing illnesses (mean 1,97; *p* = .05). In 31 European countries the old dependency ratio increases on average with 3 points between 2006 and 2014 (with highest increase in Czech Republic, Finland, Malta, Denmark, Sweden, and Netherlands) the life expectancy over 2 years on average (with highest increase in Estonia, Latvia. Lithuania, and Slovak Republic), the percentage of GDP spent on social protection with 3% (with highest increase in Greece, Spain, Finland, Ireland, Cyprus, Denmark, and Netherlands), and the percentage of long-standing illnesses increased with almost 2% (with highest increase in Austria, Estonia, and Portugal). No significant mean differences between 2006 and 2014 are found for % of GDP spent in long-term care, aggregate replacement ratio, the % of very good subjective health, and % of unmet needs in health care.

Regression analysis, with life satisfaction of older citizens in 2013 as dependent variable and mean differences in the eight indicators (2006–2014) as independent variables, shows that four indicators statistically significantly contribute to explaining the level of life satisfaction in older citizens, explaining 38% of the variance (see Table 6).

Low life satisfaction of older citizens (65 years and over) in 2013 occurs in countries, where life expectancy decreased as well as financial means for social protection and long-term care. In countries, where the percentage of unmet needs in health care between 2006 and 2014 increased, older citizens show low life satisfaction in 2013.

## 4. Discussion

Life satisfaction of older citizens in Europe is significantly decreased in 2013 as compared to younger age groups. Such a difference has not been found before, that is, in 2007 or 2003 [20, 35]. It is interesting to note the overall tendency that life satisfaction decreased in western European countries and increased in central-eastern European countries, but it seems that in the later countries younger age groups are more satisfied as compared to older ones. The former found that U-shape between age and life satisfaction (i.e., older citizens' life satisfaction is relatively higher as compared to middle age citizens and about the same as in younger citizens) does not apply for most European countries in 2014. There are exceptions like in Denmark, Ireland, Norway, Sweden, and Switzerland where life satisfaction of citizens between 65 and 74 years is higher than young age groups. However, in four of these countries, all with a high life expectancy at birth above 81 years, life satisfaction of citizens of 75 years and older decreased. So, policy measures taken between 2006 and 2014 could have affected life satisfaction of older citizens negatively. A different pattern in life satisfaction scores among the oldest is formerly noticed [15] but not understood yet. Probably, the most vulnerable old citizens suffer first from policy measures, which reduce welfare and health services.

The different patterns between age and life satisfaction in different countries indicate that not only individual factors could explain such variations. Longitudinal data of individual old citizens show that disfavoured living conditions (housing and neighbourhoods) are related to lower life satisfaction [1]. Therefore, it is important to look for the dynamics of these conditions on aggregate level to investigate the influence of policy measures on life satisfaction as stated in the third quality of life survey in Europe [20]. Our study shows that significant changes in societal indicators, related to the ageing of the population in combination with the economic crisis, occurred in European countries between 2006 and 2014. Relatively less investment in social protection and long-term care affected negatively life satisfaction of older citizens as did decline in quality of care (low increase in life expectancy and increase of unmet needs). The second quality of life survey in Europe showed that material deprivation and health status were the most important influences on life satisfaction at individual level [35]. Our outcomes suggest that also policy measures taken at national level affect life satisfaction of the most vulnerable citizens, like old citizens, directly. However, this applies especially in countries, which already have a back-log in economics and welfare. Many central-eastern European countries have arrears in social protection and quality of care, as compared to more prosperous north-western European countries with long-standing social welfare provisions. Therefore, in these countries, older citizens show more frequently a lower life satisfaction as compared to young and middle aged citizens.

As all studies, our study has shortcomings. Most evident is that not all, theoretically possible, indicators for societal changes could be included, because of the limited indicators in international data bases. The same goes for the time period. The time period is partly determined by the available data in specific years. Nevertheless, we have argued that the chosen years 2006 and 2014 are adequate. In 2006 a financial crisis was not discussed or visible. In 2014, the policy measures were implemented and people were confronted with the consequences, especially older citizens because of intervention in welfare and care facilities.

A strong point of the study is that the collected data are comparative, not only in time, but also in method of data collection and in validity of the measurements. For international, comparative research data from Eurostat or OECD are reliable, valid, and (mostly) free available.

Most studies on life satisfaction and ageing use individual indicators as explaining factors to understand variance in life satisfaction [20, 35]. Rather innovative is that we use aggregate indicators on national level, including 31 European countries, to understand changes in life satisfaction in older citizens.

Based on this study we conclude that life satisfaction of old citizens deteriorated related to policy measures, taken because of the economic crisis of 2008 and ageing of the population in Europe. These measures have changed various societal conditions negatively.

Nevertheless, some societal indicators show that social conditions clearly improved in some countries but others got worse. For example, the percentage of reported unmet needs decreased significantly in Bulgaria, Lithuania, Romania, Poland, and Estonia between 2006 and 2014 but it is not said that old citizens profited most. In Ireland, Greece, Italy, Iceland, and Belgium unmet needs in health care were more often reported. Based on our analysis, we believe that the rudimentary structure of health and welfare provisions in various central-eastern European countries still were too vulnerable to cope with the imposed policy measures and not because of attitudes or belief systems [18]. At the same time, it should be stated that knowledge and understanding on how societal processes and policy measures affect quality of life of citizens are limited. Theoretical development is still poor, especially when it comes to the interaction between policy measures, societal changes (including ageing of societies), and individual preferences and behaviour. International, comparative research, based on sound theoretical concepts, is strongly needed.

## Figures and Tables

**Table 1:** Mean scores life satisfaction per country in 2007 and 2013.

	Mean life satisfaction	
	2007	2013
Belgium	7.5	7.6
Bulgaria	5.0	4.8
Czech R	6.6	6.9
Denmark	8.5	8.0
Germany	7.2	7.3
Estonia	6.7	6.5
Greece	6.7	6.2
Spain	7.3	6.9
France	7.3	7.0
Croatia	6.4	6.3
Cyprus	7.1	6.2
Lithuania	6.3	6.7
Luxembourg	7.9	7.5
Hungary	5.6	6.2
Netherlands	7.9	7.8
Austria	7.0	7.8
Poland	6.9	7.3
Portugal	6.2	6.2
Romania	6.5	7.2
Slovenia	7.2	7.0
Slovakia	6.7	7.0
Finland	8.2	8.0
Sweden	8.3	8.0
UK	7.3	7.3
Iceland	—	8.0
Norway	8.2	7.9
Switzerland	—	7.6
Ireland	7.6	7.4
Italy	6.6	6.7
Latvia	6.0	6.5
Malta	7.6	7.1

**Table 2:** Mean scores on the demographic indicators old dependency ratio and life expectancy at birth per country in 2006 and 2014.

	Old dependency ratio 65+ to population 15–64 years		Life expectancy at birth	
	2006	2014	2006	2014
Belgium	26	27	79.5	81.4
Bulgaria	25	29	72.7	74.5
Czech R	20	26	76.7	78.9
Denmark	23	28	78.4	80.7
Germany	29	32	79.9	81.2
Estonia	25	28	73.2	77.4
Greece	28	32	79.9	81.5
Spain	24	27	81.1	83.3
France	25	28	80.9	82.8
Croatia	26	28	75.9	77.9
Cyprus	18	20	80.1	82.8
Lithuania	24	27	71.0	74.7
Luxembourg	21	20	79.4	82.3
Hungary	23	26	73.5	76.0
Netherlands	21	26	80.0	81.8
Austria	24	27	80.1	81.6
Poland	19	21	75.3	77.8
Portugal	26	30	79.0	81.3
Romania	22	24	72.5	75.0
Slovenia	22	26	78.3	81.2
Slovakia	16	19	74.5	77.0
Finland	24	30	79.6	81.3
Sweden	26	31	81.0	82.3
UK	24	27	79.5	81.4
Iceland	18	20	81.2	82.9
Norway	22	24	80.6	82.2
Switzerland	23	26	81.8	83.3
Ireland	16	19	79.3	81.4
Italy	30	33	81.4	83.2
Latvia	25	29	70.6	74.5
Malta	20	26	79.5	81.8

**Table 3:** Mean scores on the welfare indicators social protection expenditure as % of Gross Domestic Product (GDP), long-term care expenditure as % of GDP, and aggregate replacement ratio per country in 2006 and 2014.

	Social protection expenditure as % of GDP		% GDP long-term care expenditure		Aggregate replacement ratio	
	2006	2014	2006	2014	2006	2014
Belgium	27	30	2.1	2.6	.42	.47
Bulgaria	14	18	0.1	0.1	.37	.44
Czech R	18	20	0.2	1.4	.52	.55
Denmark	28	33	2.2	2.8	.37	.45
Germany	28	29	1.3	1.6	.46	.45
Estonia	12	15	0.2	0.3	.49	.47
Greece	24	32	—	0.1	.49	.60
Spain	20	26	0.9	0.9	.48	.60
France	30	34	1.5	1.9	.58	.69
Croatia	18	22	—	0.2	—	.40
Cyprus	17	22	0.2	0.2	.28	.39
Lithuania	13	15	0.6	1.0	.44	.45
Luxembourg	21	23	1.5	1.6	.66	.85
Hungary	22	21	0.6	0.7	.54	.62
Netherlands	26	31	3.4	4.3	.43	.50
Austria	28	30	1.3	1.6	.65	.60
Poland	20	18	0.4	0.4	.59	.63
Portugal	24	28	0.1	0.9	.59	.63
Romania	13	15	0.5	0.1	.44	.65
Slovenia	22	25	1.1	1.3	.41	.45
Slovakia	16	18	0.2	0.1	.57	.62
Finland	25	31	2.1	4.0	.47	.51
Sweden	29	30	3.5	3.5	.62	.60
UK	26	28	—	1.8	.45	.51
Iceland	21	24	1.8	1.8	.46	.49
Norway	22	25	2.1	3.3	.46	.59
Switzerland	24	27	2.0	2.8	.39	.44
Ireland	17	22	—	2.2	.38	.36
Italy	26	30	—	0.9	.58	.64
Latvia	12	14	0.2	0.5	.49	.44
Malta	17	18	—	—	.45	.56

**Table 4:** Mean scores on the health indicators subjective health, percentage of long-standing illness/health problem, and percentage of self-reported unmet needs because they were too expensive per country in 2006 and 2014.

	% Subjective health (very good)		% Long-standing illness/health problem 16 years and over		% Self-reported unmet needs, reason: too expensive, 16 years and over	
	2006	2014	2006	2014	2006	2014
Belgium	28	30	25	26	0.4	2.2
Bulgaria	21	17	30	20	16.3	4.4
Czech R	19	19	30	29	0.2	0.5
Denmark	41	27	29	27	0.2	0.4
Germany	14	17	39	39	4.2	0.6
Estonia	7	11	34	46	2.7	0.5
Greece	51	45	18	21	4.5	9.7
Spain	17	17	23	28	0.2	0.5
France	25	24	34	36	1.3	2.3
Croatia	17	25	—	31	—	1.4
Cyprus	49	45	28	34	3.0	4.6
Lithuania	6	7	34	29	4.7	0.7
Luxembourg	32	25	25	18	0.2	0.6
Hungary	13	18	35	35	1.5	2.1
Netherlands	21	23	32	35	0.1	0.4
Austria	37	32	21	37	0.4	0.1
Poland	15	17	34	38	5.6	3.1
Portugal	7	8	30	40	3.4	3.0
Romania	25	28	19	18	11.0	8.2
Slovenia	16	21	37	32	0.0	0.1
Slovakia	23	20	26	28	2.1	0.9
Finland	44	21	39	42	1.0	0.1
Sweden	34	33	35	32	0.9	0.5
UK	33	32	38	33	0.1	0.1
Iceland	50	40	25	30	0.8	3.4
Norway	28	32	31	35	0.4	0.2
Switzerland	25	33	34	36	0.9	1.0
Ireland	47	43	25	28	1.2	6.2
Italy	13	14	23	24	3.0	7.0
Latvia	3	4	36	41	11.7	10.5
Malta	31	20	20	28	1.4	0.9

**Table 5:** Pearson correlations between eight explaining variables and the difference of mean life satisfaction of citizens under 65 years versus over 64 years in 2013.

	Old dependency ratio	Life expectancy at birth	% GDP for social protection	% GDP for long-term care	Aggregate replacement ratio	% very good subjective health	% long standing illness	% unmet needs health care
Difference in life satisfaction 16–64 versus 65+	−.114	.608	.463	.689	−.242	.478	.149	−.315
Significance level	.542	.000	.009	.000	.190	.007	.423	.084

**Table 6:** Regression analysis and enter method, between changes in independent variables between 2006 and 2014 and life satisfaction of citizens of 65 years and older in 2013 as dependent variable in 31 European countries.

Life satisfaction of older citizens 65+ in 2013	Unstandardised coefficients		Standardised coefficients	*t*	Significance
	B	Standard error	Beta		
Constant	8.699	.696		12.491	.000
Difference in old dependency ratio 2006–2014	−.079	.116	−.120	−.685	.501
Difference in life expectancy 2006–2014	−.521	.225	−.368	−2.318	.030
Difference in % GDP social protection 2006–2014	−.188	.082	−.386	−2.292	.032
Difference in % GDP long-term care 2006–2014	.776	.332	.378	2.335	.029
Difference in aggregate replacement ratio 2006–2014	−.406	2.693	−.025	−.151	.882
Difference in % subjective health as very good 2006–2014	−.042	.025	−.275	−1.682	.107
Difference in % long standing illnesses 2006–2014	−.042	.032	−.220	−1.326	.199
Difference in % unmet needs in health care 2006–2014	.173	.053	.515	3.281	.003
